# The effect of a chemotherapy drug cocktail on myotube morphology, myofibrillar protein abundance, and substrate availability

**DOI:** 10.14814/phy2.14927

**Published:** 2021-07-01

**Authors:** Stephen Mora, Olasunkanmi A. J. Adegoke

**Affiliations:** ^1^ School of Kinesiology and Health Science and Muscle Health Research Centre York University Toronto Ontario Canada

**Keywords:** cachexia, chemotherapy, protein synthesis, skeletal muscle

## Abstract

Cachexia, a condition prevalent in many chronically ill patients, is characterized by weight loss, fatigue, and decreases in muscle mass and function. Cachexia is associated with tumor burden and disease‐related malnutrition, but other studies implicate chemotherapy as being causative. We investigated the effects of a chemotherapy drug cocktail on myofibrillar protein abundance and synthesis, anabolic signaling mechanisms, and substrate availability. On day 4 of differentiation, L6 myotubes were treated with vehicle (1.4 μl/ml DMSO) or a chemotherapy drug cocktail (a mixture of cisplatin [20 μg/ml], leucovorin [10 μg/ml], and 5‐fluorouracil [5‐FLU; 50 μg/ml]) for 24–72 h. Compared to myotubes treated with vehicle, those treated with the drug cocktail showed 50%–80% reductions in the abundance of myofibrillar proteins, including myosin heavy chain‐1, troponin, and tropomyosin (*p* < 0.05). Cells treated with only a mixture of cisplatin and 5‐FLU had identical reductions in myofibrillar protein abundance. Myotubes treated with the drug cocktail also showed >50% reductions in the phosphorylation of AKT^Ser473^ and of mTORC1 substrates ribosomal protein S6^Ser235/236^, its kinase S6K1^Thr389^ and eukaryotic translation initiation factor 4E‐binding protein 1 (all *p* < 0.05). Drug treatment impaired peptide chain initiation in myofibrillar protein fractions and insulin‐stimulated glucose uptake (*p* = 0.06) but increased the expression of autophagy markers beclin‐1 and microtubule‐associated proteins 1A/1B light chain 3B (*p* < 0.05), and of apoptotic marker, cleaved caspase 3 (*p* < 0.05). Drug treatment reduced the expression of mitochondrial markers cytochrome oxidase and succinate dehydrogenase (*p* < 0.05). The observed profound negative effects of this chemotherapy drug cocktail on myotubes underlie a need for approaches that can reduce the negative effects of these drugs on muscle metabolism.

## Graphical abstract

We showed that a chemotherapy drug cocktail suppressed myofibrillar protein abundance and synthesis, and reduced insulin‐stimulated glucose transport and the expression of an amino acid transporter. Drug treatment also increased the expression of markers of autophagy and apoptosis. Thus, the adverse effects of these drugs on muscle cells are linked to reduced substrate availability.

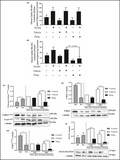

## 1 INTRODUCTION

Cachexia is a condition that affects greater than nine million patients with chronic diseases such as cancer, chronic heart failure, and kidney disease (Haehling & Anker, 2010). Cachexia leads to wide scale fatigue and weakness, associated with decreases in quality of life and increased mortality and morbidity. It is a wasting disorder characterized by severe loss of body weight and depletion of muscle mass and adipose tissue (Ozola Zalite et al., 2015; Thoresen et al., 2013).

Maintenance of skeletal muscle mass is dependent on an intricate balance between protein synthesis and protein degradation. These two processes are highly regulated by multiple signaling events and pathways. For example, activation of the insulin receptor substrate 1 (IRS‐1)/protein kinase B (AKT)/mammalian (mechanistic) target of rapamycin complex 1 (mTORC1) pathway is vital for skeletal muscle growth and inhibition of skeletal muscle protein degradation (Adegoke et al., 2012; Bodine, Stitt, et al., 2001). Stimulation of either the ubiquitin proteasome pathway or autophagy/lysosomal pathway induces protein degradation within skeletal muscle (Bodine, Latres, et al., 2001; Sandri, 2013). Activation of either pathway is regulated by factors including the forkhead box protein O (FOXO) (Lecker et al., 2004; Senf et al., 2010). In addition, mitochondrial homeostasis has been linked to muscle wasting (Ballarò et al., 2019). Damaged mitochondria activate the AMP‐activated protein kinase pathway (Zhao et al., 2016), leading to an upregulation of FOXO transcription factors and atrogin‐1 (Sandri et al., 2004). Mitochondria derived reactive oxygen species also activate FOXO and nuclear factor kappa‐light‐chain enhancer of activated B cells (NF‐KB) signaling mechanisms (Dodd et al., 2010) leading to muscle atrophy.

Since lean body mass is a determinant of chemotherapy dose (Ali et al., 2016), patients with reduced muscle mass face the possibility of reduced treatment efficacy, increased risk of drug toxicity, and reduced chance of disease‐free survival (Antoun et al., 2010; Jung et al., 2014). Therefore, a better understanding of the mechanism of disease‐and/or treatment‐induced cachexia may lead to the development of interventions that can limit the debilitating effects of this syndrome.

Although available evidence suggests an association between chemotherapy treatment and cachexia, the mechanisms of effects of chemotherapy‐induced cachexia are not completely understood. Cisplatin, a platinum‐containing chemotherapy drug used in the treatment of testicular, ovarian, head, lung, and neck cancers (Dasari & Tchounwou, 2014), increases mRNA expression of muscle atrophy gene F‐box, muscle RING finger‐1, and FOXO3 in vivo (Sakai et al., 2014). In addition, administration of cisplatin in muscle cells activates NF‐κB (Damrauer et al., 2018), a proteolysis‐inducing signaling protein (Cai et al., 2004). Other drugs, such as 5‐fluorouracil (5‐FLU), a component of folfiri and folfox chemotherapy regimens that are used in the treatment of metastatic colorectal cancer (Douillard et al., 2000), upregulate p38 mitogen activated protein kinases resulting in weight loss, muscle loss, and reductions in mitochondrial content (Barreto, Waning, et al., 2016). In addition, 5‐FLU administration leads to body weight loss, anorexia, and reductions in nitrogen balance in healthy and tumor‐bearing mice (Le Bricon et al., 1995). Clinically, 5‐FLU is often combined with leucovorin (a folic acid derivative) to treat colorectal cancer (Gramont et al., 2000). Mechanistically, 5‐FLU acts as an anti‐cancer agent by inhibiting a key enzyme in DNA biosynthesis, thymidylate synthase (Peters et al., 2002). However, 5‐FLU alone is metabolized quickly and only stays in circulation for a short period of time. In combination with leucovorin, the inhibition of thymidylate synthase by 5‐FLU is enhanced (Peters et al., 1992), leading to a greater tumor response rate (Piedbois et al., 1992) and survival in patients with advanced colorectal cancer (Poon et al., 1989). Other common chemotherapy drugs, such as doxorubicin, decrease muscle protein synthesis (Nissinen et al., 2016) and reduces insulin‐stimulated glucose uptake (de Lima Junior et al., 2016), both of which may lead to cachectic symptoms.

Previous studies have examined the effect of individual chemotherapy drugs such as cisplatin on muscle wasting (Damrauer et al., 2018) or of 5‐FLU on body weight loss (Sotos et al., 1994). However, chemotherapy drugs are most often administered or studied as cocktails (Ballarò et al., 2019; Barreto, Waning, et al., 2016; Douillard et al., 2000). In addition, previous studies have looked at the effect of chemotherapy drugs on total muscle proteins (Barreto, Waning, et al., 2016). However, it is not clear whether myofibrillar proteins are specifically affected by these chemotherapy drugs. Lastly, although chemotherapy drugs reduce insulin‐stimulated glucose uptake (de Lima Junior et al., 2016), it is not clear whether amino acid transport is similarly affected, an important point given the significance of amino acids in regulating muscle protein metabolism and mass. Therefore, the purpose of this study was to investigate the effects of a combinatory chemotherapy drug cocktail (cisplatin, 5‐FLU, and leucovorin) on myotube morphology, myofibrillar protein abundance, and synthesis, and the modulation of anabolic signaling pathways. In addition, we examined the effects of these drugs on measures of insulin sensitivity and substrate transport into muscle cells.

## 2 MATERIALS AND METHODS

### 2.1 Cell culture

L6 skeletal myoblasts (American Type Culture Collection) were cultured in AMEM supplemented with 10% FBS and 1% antibiotic‐antimycotic reagents at 37°C and 5% CO_2_. Once confluent (day 0, D0), cells were shifted into AMEM supplemented with 1% antibiotic‐antimycotic reagents and 2% horse serum to induce myotube differentiation. Medium was replaced every other day until day 4 of differentiation (D4). Myoblasts at D0 and myotubes at D4 were harvested and used as controls. In separate plates, D4 myotubes were treated with fresh differentiation medium in combination with either a chemotherapy drug cocktail ([20 μg/ml cisplatin, 50 μg/ml 5‐FLU, and 10 μg/ml leucovorin], Sigma Aldrich) or vehicle ([1.4 μl/ml DMSO], Sigma Aldrich). Following treatment initiation on D4, myotubes remained under respective vehicle or drug treatments for 24, 48, or 72 h (corresponding to D5, D6, or D7 of differentiation, respectively). Although this cocktail is not used in clinics, cisplatin, and 5‐FLU are extensively used together to treat cancers of the esophagus, head, neck, and anus (Hitt et al., 2002; Psyrri et al., 2004; Rao et al., 2015). In addition, leucovorin is often used in combination with 5‐FLU (Gramont et al., 2000; Kemeny et al., 2004). Therefore, we were interested in studying the combined effects of the three drugs on myotubes. For experiments in which we used C2C12, myoblasts were cultured in DMEM supplemented with 10% FBS and 1% antibiotic‐antimycotic reagents at 37°C and 5% CO_2_. Once confluent, cells were shifted into DMEM supplemented with 1% antibiotic‐antimycotic reagents and 2% horse serum. On D4, C2C12 myotubes were treated with vehicle or chemotherapy drug cocktail as described for L6 myotubes. Compared to L6, C2C12 cells are derived from mouse and exhibit differences in glucose transport (Sarabia et al., 1990) and mitochondrial respiration (Robinson et al., 2019). Therefore, we examined whether findings on the effect of the chemotherapy drug cocktail in L6 myotubes could be replicated in C1C12 myotubes.

### 2.2 Antibodies

Antibodies to myosin heavy chain‐1 (MHC‐1, MF20), troponin (JLT12), and tropomyosin (CH1) were obtained from Developmental Hybridoma. Antibodies to phosphorylated (p) ribosomal protein S6^Ser235/236^ (S6) (#4858), its kinase (S6K1^thr389^) (#9234), p‐Akt^Ser473^ (#4060), p‐eukaryotic translation initiation factor 4E‐binding protein 1 (4EBP1^Thr37/46^) (#2855), SNAT1 (sodium‐coupled neutral amino acid transporter 1, #36057), p‐IRS1^Ser612^ (#3203), total (t)‐IRS1 (#2382), p‐glycogen synthase (GS)^Ser641^ (#47043), Beclin‐1 (#3738), microtubule‐associated proteins 1A/1B light chain 3B (LC3B) (#3868), sqstm1/p62 (#5114), caspase‐3 (#9662), caspase‐7 (#12827), succinate dehydrogenase (SDHA) (#11998), cytochrome oxidase (COXIV) (#4850), and GAPDH (#2118) were purchased from Cell Signaling Technology. Anti‐ubiquitin antibody (#sc‐8017) was purchased from Santa Cruz Technology and γ‐tubulin (#T6557) from Sigma Aldrich.

### 2.3 Immunofluorescence microscopy

Following treatments, myotubes cultured on cover slips were fixed in 4% paraformaldehyde solution (PFA in PBS), permeabilized with Triton Solution (0.03% Triton X‐100 in PBS) and incubated in a blocking solution (10% horse serum in PBS) for 1 h at 37°C. Myotubes were then exposed overnight at 4°C in a diluted MHC primary antibody solution (2.5 μg/ml of MHC in 1% bovine serum albumin [BSA] in PBS). The following day, cells were washed and exposed to a diluted Texas Red anti‐mouse IgG secondary antibody solution (1:100 with 1% BSA in PBS) before 4′,6‐diamidino‐2‐phenylindole (DAPI) staining (for nuclei) and cover slip mounting on microscope slides. Slides were then imaged using the EVOS FL Auto microscope (Life Technologies) along with the EVOS FL Auto program for maintaining acquisition settings in all experimental treatments. Images were quantified as previously described (Rocco et al., 2019). Briefly, all images were transformed into an 8‐bit gray scale image and mean gray value of each sample was measured within a 0–255 range using Image J.

### 2.4 Western blotting

Western blotting was carried out as previously described (Jeganathan et al., 2014; Zargar et al., 2011). Briefly, the pierce BCA protein assay kit (Thermo Fisher, #23225) was used to determine protein concentration. Equal amounts of protein (~25 µg) were separated on 10% or 15% SDS‐PAGE gels and transferred onto polyvinylidene difluoride membranes (0.2 µM, BIO‐RAD). Primary and secondary antibody incubation, imaging, and quantification were all done as previously described (Jeganathan et al., 2014; Zargar et al., 2011). In short, after overnight incubation in primary antibodies, membranes were washed 3 × 5 min in TBST and then incubated in HRP‐conjugated anti mouse or anti‐goat IgG, depending on the source of the primary antibodies. Following another 3 × 5 min washes in TBST, HRP chemical luminescent substrate was added to each membrane and BIO‐RAD ChemiDoc XRS+ was used for signal visualization.

### 2.5 Peptide chain initiation

At 24 and 48 h following the initiation of drug treatment, myotubes were incubated in a protein synthesis labeling mix (1X DMEM lacking methionine and cysteine, 2 μCi/ml of ^35^S methionine/cysteine [Perkin Elmer, NEG072007MC] and 2% dialyzed FBS) for 1 h. Next, cells were washed 5X in ice‐cold PBS and harvested with lysis buffer (1 mM EDTA, 2% sodium dodecyl sulfate [SDS], 25 mM Tris‐HCl, pH 7.5, 1 mM DTT, and 10 μl/ml of each of protease and phosphatase inhibitor cocktails). Following 10% SDS–PAGE, gels were stained with Coomassie Bright Blue Solution for 1 h (0.1 g Coomassie Blue R‐250 dissolved in 100 ml of 50% methanol, 5% acetic acid, and 45% double distilled [DDH_2_O] solution). Next, gels were washed 4X in destaining solution (60% DDH_2_O, 20% methanol, 10% glacial acetic acid) and left overnight at 4°C in destaining solution. The following day, a Coomassie Blue Blot (CBB) image of the stained membranes was taken with the Typhoon FLA 9500 imager. Gels were then dried for 1 h using a Model 583 Gel Dryer from BIO‐RAD and placed inside an autoradiography cassette (Fisher Scientific: FBCA 810) and exposed to a 20 × 25 cm phospho imaging screen (Fujifilm #An28956475). Following the exposure period, the phosphor imaging screen was scanned using Typhoon FLA 9500 imager. Previous studies have also used ^35^S methionine/cysteine incorporation as an analysis of peptide chain initiation (Kim et al., 2005; Tominaga et al., 2005).

### 2.6 Myotube fractionation

Our procedure was modified from a previous study (El Naggar et al., 2004). Following treatments, myotubes were trypsinized and collected into 15 ml‐test tubes. Cells were centrifuged (0.4 g, 5 min, room temperature) and resuspended in PBS. They were then centrifuged as before and resuspended in 500 μl of buffer1 (1% Triton in PBS) supplemented with protease (10 μl/ml) and phosphatase inhibitor (10 μl/ml) cocktails and EDTA (1 mM). Of the 500 μl, 100 μl was extracted for determination of protein concentration and also used as the load fraction, while the remaining 400 μl was centrifuged (0.1 g, 5 min, 4°C). The supernatant was extracted and labeled as the sarcoplasmic fraction. The pellet (myofibrillar fraction) was resuspended in 300 µl of buffer 2 (10 μl/ml of protease inhibitor in myofibrillar protein isolation buffer [300 mM NaCl, 100 mM NaH_2_PO_4_, 50 mM NaH_2_PO_4_, 10 mM Na_4_P_2_O_7_, 10 mM EDTA, 1 mM MgCl_2_, pH 6.5, 0.1% mercaptoethanol]) and left on ice for 40 min. The pellet was centrifuged (0.1 g, 5 min, 4°C) and resuspended in filament buffer (double distilled water, 0.1% betamercaptoethanol, 1 mM EDTA) overnight on ice. The following day, pellet was centrifuged (0.1 g, 30 min, 4°C), resuspended in filament buffer and centrifuged again (0.1 g, 3 min, 4°C). Finally, the pellet was dissolved in 50 μl of 1X sample buffer. Since MHC‐1 and GAPDH are myofibrillar and sarcoplasmic proteins respectively, we expected to see MHC‐1 only in the myofibrillar fraction and GAPDH only in the sarcoplasmic fraction. Gamma tubulin was used as a loading control.

### 2.7 Glucose transport

Following treatments, myotubes were incubated in starvation medium (complete starvation medium, free of amino acids and serum) for 3 h. They were then incubated with or without 100 nM of insulin for 20 min, rinsed twice with 2X HEPES (4‐(2‐hydroxyethyl) piperazine‐1‐ethanesulfonic acid) buffered saline and then incubated for 5 min at 37°C in 300 μl of transport solution (HEPES buffer, 10 μM 2‐deoxyglucose, 0.5 μCi/ml [^3^H]‐2‐ deoxyglucose). Samples were harvested and processed as previously described (Jeganathan et al., 2014).

### 2.8 Statistical analyses

Quantification data for immunoblot analyses were adjusted by their corresponding gamma tubulin values and then normalized to the 24 h vehicle group. For some experiments, separate target proteins were imaged in parallel and corrected to similar gamma tubulin values. All graphs were drawn using Prism Software Version 7. Unpaired *t*‐test with a Welch correction was used to analyze immunofluorescence results and to compare D0 myoblasts to D4 myotubes. Two‐way ANOVA was used to analyze treatment and time combination, followed by a Tukey's post hoc Test. Differences were found to be significant when *p* < 0.05. All results were expressed as means ± SEM of at least three independent experiments (biological replicates).

## 3 RESULTS

### 3.1 Myotube morphology and abundance of myofibrillar proteins are negatively regulated by a chemotherapy drug cocktail

**FIGURE 1:**
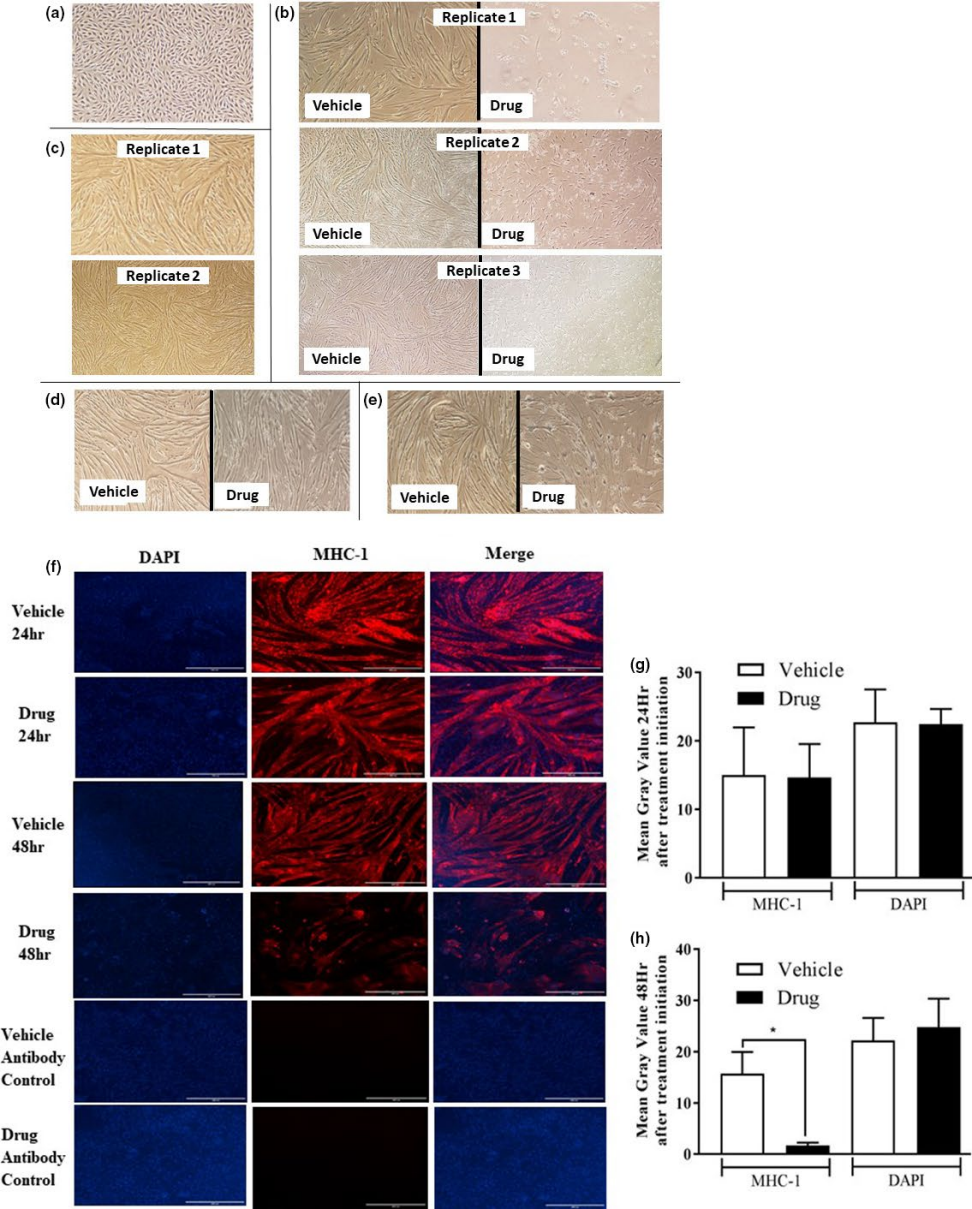
Myotube morphology and abundance of myofibrillar proteins are negatively regulated by a chemotherapy drug cocktail. Using the Nikon eclipse TS100 with the 10× objective lens, light microscope images of myotubes in the different treatment groups are shown. (a) L6 myoblasts (Day 0) at the onset of differentiation prior to the application of either vehicle (1.4 μl/ml DMSO) or chemotherapy drug cocktail (20 μg/ml cisplatin, 50 μg/ml 5‐fluorouracil and 10 μg/ml leucovorin) treatment. (b) Following the initiation of drug treatment at the onset of differentiation, myoblasts treated with the drug cocktail (right) were unable to form myotubes compared to vehicle (left). Representative images from each of three different experiments are shown. In a separate experiment, myoblasts at D0 and myotubes at D4 were harvested and used as controls. On day 4 of differentiation (c, untreated myotubes), myotubes were treated with vehicle or the chemotherapy drug cocktail and observed 24 (d) and 48 h (e) following the initiation of treatment. (f) Immunofluorescence detection of myosin heavy chain‐1 (MHC‐1) 24 and 48 h following the initiation of drug treatment in myotubes stained with DAPI (Bar, 400 μm). Vehicle and drug antibody control represents myotubes under their respective treatments incubated with primary, but no secondary antibody. Quantified mean gray value of MHC‐1 immunoreactive staining and DAPI nuclear staining in vehicle and drug‐treated myotubes 24 (g) and 48 h (h) following the initiation of drug treatment. Data are mean ± SEM, *n* = 4 independent experiments (biological replicates), with at least three technical replicates in each treatment group for each of the independent experiments, *p < 0.05. DAPI, 4′,6‐diamidino‐2‐phenylindole

**FIGURE 2:**
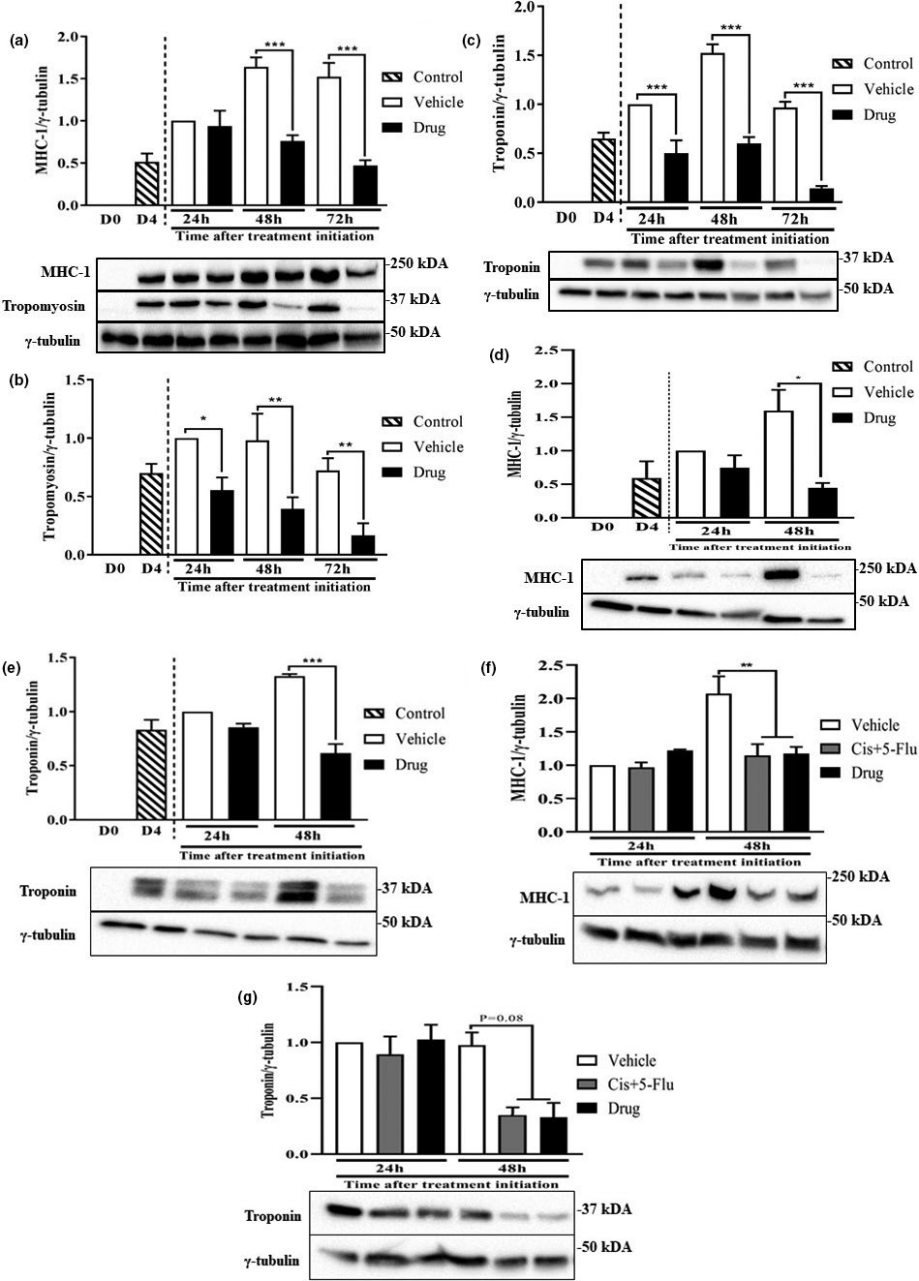
Myotubes treated with the chemotherapy drug cocktail exhibit reductions in myofibrillar protein abundance. L6 myotubes were differentiated and treated as described in Figure 1. Control represents the group that received no vehicle or chemotherapy drug treatment. Samples were harvested 24, 48, and 72 h following initiation of drug treatment and immunoblotted for MHC‐1 (a), tropomyosin (a, b), and troponin (c). MHC‐1, tropomyosin, and the corresponding γ‐tubulin (loading control) were imaged on the same membranes. C2C12 myotubes were treated with vehicle or a chemotherapy drug cocktail as described for L6 myotubes. Myotubes were harvested and blotted for MHC‐1 (d) and troponin (e) 24 and 48 h following the initiation of drug treatment. In (f, g) myotubes were treated with the chemotherapy drug cocktail described earlier (drug) or a cocktail composed of 20 μg/ml cisplatin and 50 μg/ml 5‐fluorouracil (Cis + 5‐Flu) and blotted for MHC‐1 (f) and troponin (g). Data are mean ± SEM, *n* = 3–4 independent experiments, except in (g) where for technical reasons *n* = 2 because we could not obtain usable troponin blot for one of the three experiments. *p < 0.05, **p < 0.01, ***p < 0.001. MHC, myosin heavy chain‐1

In an initial experiment, we examined the effects of the drug cocktail on myotube formation. At the onset of differentiation (Figure 1a), D0 myoblasts were treated with DMSO (Vehicle) or the chemotherapy drug cocktail (Drug). By D4 of differentiation, myoblasts treated with the drug cocktail did not form myotubes (Figure 1b, right) compared to vehicle (Figure 1b, left). We then investigated the effect of the drug cocktail on myotubes differentiated for 4 days. (Figure 1c). Twenty‐four hours (Figure 1d) and 48 h (Figure 1e) after the initiation of drug treatment, abnormalities in myotube morphology were observed. Twenty‐four hours following drug treatment, immunofluorescence analysis showed no substantial difference in MHC‐1 staining between groups (Figure 1f,g). However, at 48 h, treated myotubes exhibited significant reductions (~85%) in MHC‐1 staining (Figure 1f,h). Consistent with this data, at 48 and 72 h following initiation of drug treatment, myotubes treated with the drug cocktail showed >50% reductions in MHC‐1 protein abundance (Figure 2a). In addition, >50% reductions in protein abundance were found for tropomyosin and troponin 24, 48, and 72 h following initiation of drug treatment (Figure 2a–c). Similar observations were made in C2C12 myotubes treated with the drug cocktail for MHC‐1 (Figure 2d) and Troponin (Figure 2e). Removal of leucovorin from the drug cocktail did not change the effect of the cocktail on MHC‐1 (Figure 2f) or troponin (Figure 2g) abundance.

### 3.2 Anabolic signaling and protein synthesis are decreased following treatment of myotubes with a chemotherapy drug cocktail

**FIGURE 3:**
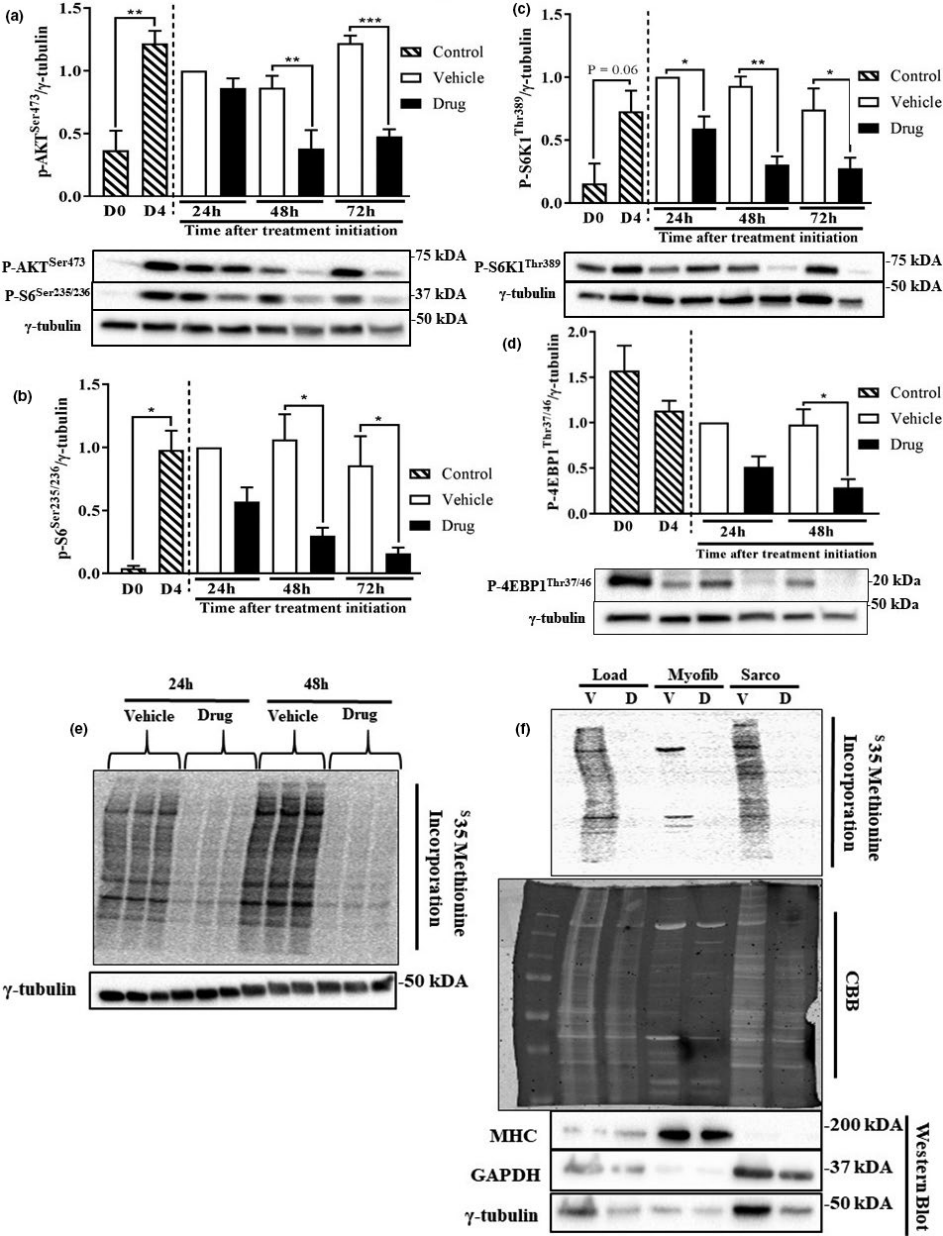
Anabolic signaling and protein synthesis are decreased following treatment of myotubes with a chemotherapy drug cocktail. L6 myotubes were differentiated and treated as described in Figure 1. Phosphorylation of AKT^Ser473^ (a), S6^Ser235/236^ (a, b), S6K1^Thr389^ (c), and 4EBP1^Thr37/46^ (d) were detected by western blotting in vehicle and drug‐treated myotubes 24, 48, and 72 h following initiation treatments. P‐AKT^Ser473^, S6^Ser235/236^, and the corresponding γ‐tubulin (loading control) were imaged on the same membranes. (e) Autoradiograph showing peptide chain initiation, using ^35^S methionine/cysteine, 24 and 48 h following initiation of treatments. (f) Autoradiograph showing peptide chain initiation in myofibrillar and sarcoplasmic protein fractions at 48 h of treatment. Protein loading (CBB) and fractionation efficiency (Western Blot) are shown. Data are mean ± SEM, *n* = 3 for p‐S6K1 and p‐S6, *n* = 4 for p‐AKT and p‐4EBP1, *p < 0.05, **p < 0.01, ***p < 0.001. CBB, Coomassie Blue Blot

Due to the decreases in myofibrillar protein content, we examined proteins involved in mTORC1 signaling, a master regulator of muscle protein synthesis and mass (Adegoke et al., 2012; Bodine, Stitt, et al., 2001). In the absence of any treatment, phosphorylation (p) of AKT^Ser473^ (mTORC1 upstream activator), S6^Ser235/236^ (ribosomal protein), and S6K1^Thr389^ (mTORC1 substrate) were all increased in D4 myotubes compared to D0 myoblasts (Figure 3a–c). Myotubes treated with the drug cocktail showed significant reductions (>50% for all) in the phosphorylation of AKT^Ser473^, S6^Ser235/236^ and S6K1^Thr389^ 48 and 72 h after the initiation of drug treatment (Figure 3a–c). Only phosphorylation of S6K1^Thr389^ was found to be decreased (40%) at the 24 h time point (Figure 3c). In addition, phosphorylation of 4EBP1^Thr37/46^ was significantly reduced (75%) 48 h after the initiation of drug treatment (Figure 3d). Due to the observed changes in the protein content and mTORC1 signaling, we next measured ^35^S amino acid incorporation into peptides, a measure of peptide chain initiation. At 24 and 48 h, myotubes treated with the drug cocktail showed a decrease in peptide chain initiation (Figure 3e). Furthermore, peptide chain initiation in myofibrillar and sarcoplasmic fractions was also reduced at the 48 h time point. Equal protein loading (CBB) and efficiency of fractionation (using MHC and GAPDH as markers) are shown in Figure 3f.

### 3.3 Glucose transport and the expression of an amino acid transporter are altered following chemotherapy drug treatment

**FIGURE 4:**
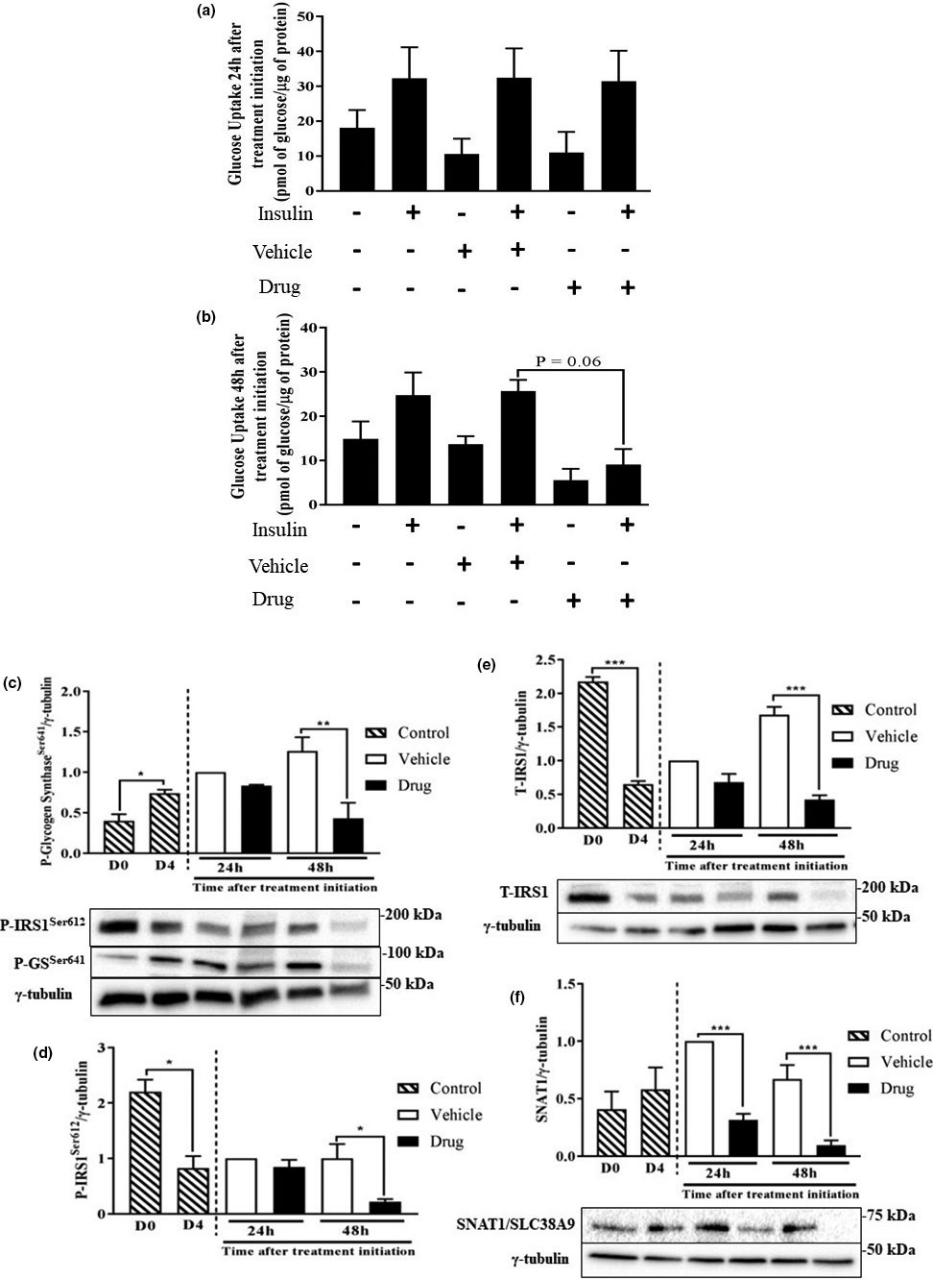
Glucose transport and the expression of an amino acid transporter are altered following chemotherapy drug treatment. L6 myotubes were differentiated and treated as described in Figure 1. At 24 (a) and 48 h (b) basal and insulin‐stimulated glucose uptake in control, vehicle and chemotherapy drug‐treated myotubes was examined. Glucose transport was expressed as picomole per μg of protein. Using western blotting analysis, p‐GS^Ser641^ (c), p‐IRS1^Ser612^ (c, d), t‐IRS1 (e) and SLC38A9/SNAT1 (f) were detected in the different groups 24 and 48 h following initiation of treatment. P‐GS^Ser641^, p‐IRS1^Ser612^, and γ‐tubulin (loading control) were imaged on the same membranes. Data are mean ± SEM, *n* = 3, *p < 0.05, **p < 0.01, ***p < 0.001

Due to the finding that phosphorylation of AKT^Ser473^ was reduced in myotubes treated with the drug cocktail (Figure 3a) and to examine whether the effects of the drug cocktail on myotubes is linked to substrate availability, we next investigated insulin‐stimulated glucose uptake. There were no differences in either basal or insulin‐stimulated glucose uptake across treatments 24 h after initiation of drug treatment (Figure 4a). At the 48 h time point, there was a trend toward a significant reduction (*p* = 0.06) in insulin‐stimulated glucose uptake (Figure 4b). To study other indicators of glucose metabolism, we examined phosphorylation of GS, which when phosphorylated on Ser641 would suggest reduced glycogen synthesis (Jensen et al., 2012). P‐GS and P‐IRS1 were significantly increased (~50%) and decreased (~60%) respectively, in D4 myotubes compared to D0 myoblasts (Figure 4c,d). Surprisingly, GS^Ser641^ (Figure 4c) and IRS1^Ser612^ (Figure 4c,d) were decreased (60% and 70%, respectively) at 48 h following the initiation of drug treatment. We note that total IRS‐1 level was also reduced (Figure 4e). In addition, there were significant reductions (>50% at all time points) in protein expression of the amino acid transporter SLC38A9/SNAT1 at 24 and 48 h (Figure 4f).

### 3.4 Ubiquitinated proteins, abundance of autophagy markers, and apoptosis in myotubes treated with the chemotherapy drug cocktail

**FIGURE 5:**
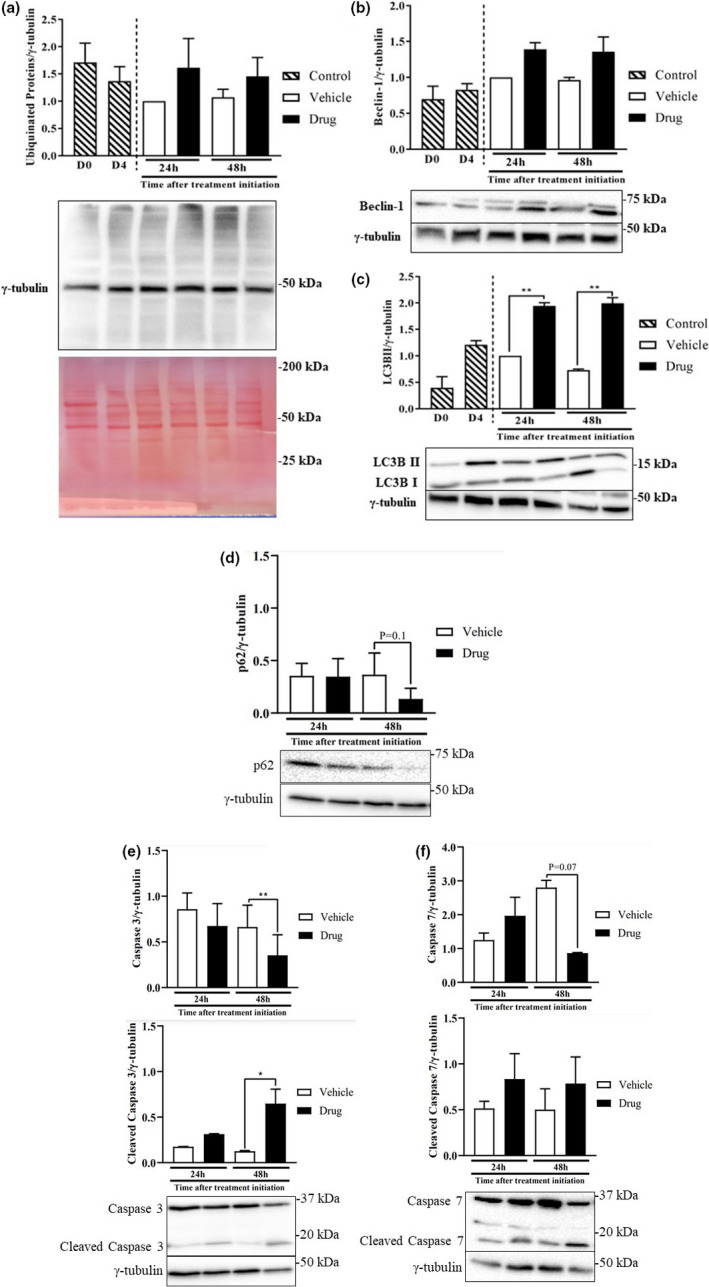
Ubiquitinated proteins and the abundance of autophagy/apoptosis markers in myotubes treated with the chemotherapy drug cocktail. L6 myotubes were differentiated and treated as described in Figure 1. Ubiquitin (a), beclin‐1 (b), LC3BII (c), and p62 (d) were measured in the different groups 24 and 48 h following initiation of treatments. Total caspase 3 (e), caspase 7 (f) and their respective cleaved fragments were also measured. Data are mean ± SEM, *n* = 3, except the data for p62 for which, due to technical reasons, *n* = 2, *p < 0.05, **p < 0.01

Due to the finding that mTORC1 activity and peptide chain initiation were decreased (Figure 3), we hypothesized that myotubes treated with the drug cocktail would be in a state of elevated catabolism. However, there was no significant effect on the abundance of ubiquitinated proteins (Figure 5a), a marker of activation of the ubiquitin dependent proteolytic pathway (Sandri, 2013), but there was a trend for increased beclin1, a marker of the autophagy/lysosomal proteolytic system (Kang et al., 2011), at 24 and 48 h (*p* = 0.1 for both time points) (Figure 5b). Significant increases (~2‐fold) were found for LC3BII (Figure 5c), another marker of autophagy. Consistent with the induction of autophagy, there was a trend for reduced p62 abundance at 48 h in myotubes treated with the drug cocktail (Figure 5d). At 48 h of treatment, total, and cleaved caspase 3 were decreased (~50%) and increased (~3.5‐fold), respectively, in drug‐treated myotubes (Figure 5e). Although there was a trend for decreased caspase 7, cleaved caspase 7 was not different between groups (Figure 5f).

### 3.5 Markers of mitochondrial abundance are reduced following chemotherapy drug treatment

**FIGURE 6:**
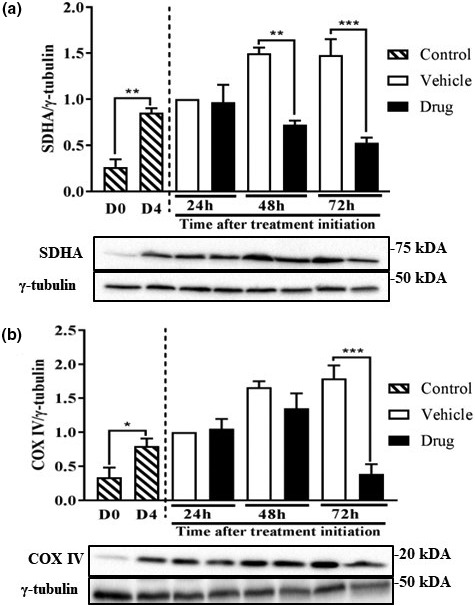
Markers of mitochondrial abundance are reduced following chemotherapy drug treatment. L6 myotubes were differentiated and treated as described in Figure 1. SDHA (a) and COXIV (b) were measured at the indicated times in the different treatment groups. Data are mean ± SEM, *n* = 4, *p < 0.05, **p < 0.01, ***p < 0.001. COXIV, cytochrome oxidase; SDHA, succinate dehydrogenase

Since mitochondria dysfunction has been implicated in the prognosis of muscle wasting (Ballarò et al., 2019), we measured mitochondrial content markers. SDHA and COXIV were both increased in D4 myotubes compared to D0 myoblasts, likely due to increased energy demands. Compared to myotubes treated with vehicle, those treated with the drug cocktail showed significant reductions in SDHA at both 48 (50%) and 72 h (60%) after the initiation of drug treatment (Figure 6a). Significant reductions (75%) were also found for COXIV (Figure 6b).

## 4 DISCUSSION

We showed that treatment of myotubes with the chemotherapy agents cisplatin, 5‐FLU, and leucovorin led to abnormal myotube morphology, impaired protein metabolism, and altered substrate availability. These effects appeared to be mediated by cisplatin and 5‐FLU combination (normally used for the treatment of diverse cancers), as the effects on myofibrillar protein abundance were identical whether or not leucovorin was added. Although some earlier studies have examined the effects of chemotherapy drugs on muscle metabolism (Barreto, Mandili, et al., 2016; Barreto, Waning, et al., 2016), we demonstrated specific pronounced effects of this chemotherapy drug cocktail on both the abundance of myofibrillar proteins MHC‐1, troponin, and tropomyosin, and on incorporation of radiolabeled amino acids into total and (especially) myofibrillar protein fractions. These changes occurred along with reduced substrate (amino acid, glucose) availability, and insulin signaling.

Several factors may explain the altered morphology and impaired protein metabolism in these myotubes. These include reduced protein synthesis and increased proteolysis. The effect of the drug cocktail on myofibrillar protein abundance occurred in parallel with suppressed mTORC1 signaling, consistent with reduced phosphorylation of S6K1 seen in denervation‐induced muscle atrophy (MacDonald et al., 2014) and in other cachexia models (Zhang et al., 2012). The reduced mTORC1 signaling is also consistent with reduced protein synthesis in these myotubes as mTORC1 mediates anabolic signaling (Goodman, 2014). Although we did not measure proteolysis per se, we observed a small, but insignificant increase in ubiquitinated proteins in cells treated with the drug cocktail. Along the same line, drug treatment increased LC3BII and beclin1, but reduced the abundance of p62, markers of autophagy/lysosomal proteolytic system (Kang et al., 2011), a process that is regulated by mTORC1 (Kim et al., 2011). These data are consistent with increased autophagy (Yang et al., 2010), as shown in a previous study with folfiri (chemotherapy) treatment. However, only total LC3B and not LC3B II was investigated in that study (Barreto, Waning, et al., 2016). We also demonstrated reduced abundance of total caspases 3 and 7, but increased amount of cleaved caspase 3, consistent with increased apoptosis (Porter & Jänicke, 1999). There is evidence that mitochondrial imbalance is implicated in the development of muscle wasting (Ballarò et al., 2019). This is consistent with our data showing reduced level of COXIV and SDHA. Interestingly, cancer cachectic patients also show significant increases in beclin1 and LC3BII, as well as mitochondrial associated autophagy, mitophagy (Aversa et al., 2016). Together, these data point to co‐ordinated regulation of both protein synthesis and proteolysis that put drug‐treated myotubes in a catabolic state.

Reduced myofibrillar protein abundance and synthesis may also be linked to altered substrate delivery to the cells, since the substrates are needed as a source of energy (glucose) and as building blocks for protein synthesis (amino acids). Consistent with this, we observed a robust reduction in insulin‐stimulated glucose transport. The mechanism of regulation of glucose uptake in skeletal muscle is tightly regulated, involving multiple signaling proteins. Activation of IRS‐1 by the binding of insulin to the α‐subunit of its cognate insulin receptor leads to the activation of the PI3K pathway (Roques & Vidal, 1999). Acting via the phosphorylation of AKT (Franke et al., 1995), the PI3K pathway induces the translocation of glucose transporter 4 to the cell surface, thereby promoting glucose uptake. The finding of reduced glucose uptake is consistent with reduced AKT phosphorylation observed in chemotherapy drug‐treated myotubes. Interestingly, doxorubicin treatment too decreased insulin‐stimulated glucose uptake in L6 cells (de Lima Junior et al., 2016). We also observed reduced expression of a sodium‐coupled neutral amino acid transporter (SNAT1, or solute carrier family 38 member 1 [SLC38A1]). This observation is consistent with an earlier report with doxorubicin, a chemotherapy drug that not only decreases muscle protein synthesis (Nissinen et al., 2016), but also negatively affects free amino acid pools within skeletal muscle (Fabris & MacLean, 2018). Because of the roles of some amino acids (including the branched‐chain amino acids, glutamine, and arginine) in stimulating mTORC1 (Dyachok et al., 2016), chemotherapy drug‐induced impairment in amino acid transport would not only reduce substrate availability, but also suppress anabolic signaling needed for protein synthesis.

Finally, the altered morphology and impaired protein metabolism in chemotherapy drug‐treated myotubes may be linked to impaired signaling by anabolic hormones such as insulin. Along this line, we observed reduced AKT phosphorylation in response to drug treatment as has been reported by others (Fanzani et al., 2011). However, and surprisingly, IRS1 serine phosphorylation was reduced in the drug‐treated groups. This is likely related to reduced mTORC1 signaling, as this kinase complex is implicated in the inhibitory serine phosphorylation of IRS1 (Lynch & Adams, 2014; Yoon, 2016). Phosphorylation of IRS1 can lead to varied outcomes, depending on the residues that are phosphorylated. For example, serine phosphorylation of IRS‐1 usually leads to the termination of insulin signaling (Giraud et al., 2007), while tyrosine phosphorylation of IRS‐1 promotes insulin signaling (Gual et al., 2005). In addition, we found decreased serine phosphorylation of GS in the drug‐treated groups, suggestive of increased glycogen synthesis (Jensen et al., 2012). This is an unexpected finding, but one that is consistent with data showing tendency for increased muscle glycogen level in a model of cancer and chemotherapy‐induced cachexia (Pin et al., 2019). Findings related to the effect of chemotherapy on glucose metabolism are potentially critical as pre‐existing diabetes in patients receiving chemotherapy treatment can lead to both glycemic issues and an increased risk of death (Barone et al., 2008).

Limitations of this study include the fact that the chemotherapy drugs and their effects were not studied individually. Whereas others have studied the effects of individual (Damrauer et al., 2018; Sotos et al., 1994), the goal of our study was to investigate the combined effects of chemotherapy drugs, as this is the usual practice in clinical settings (Douillard et al., 2000). The drug doses we used were based on a previous study (Barreto, Waning, et al., 2016). Plasma concentrations of cisplatin in the range 0.37 to 17 µg/ml have been reported in cancer patients being treated with the drug (Panteix et al., 2002; Rajkumar et al., 2016), so the concentration we used (20 µg/ml) is not far from values reported in patients and hence the relevance of our work. The concentration of 5‐FLU that we used (50 µg/ml) is much higher than blood concentration range (0.106–3.0 µg/ml) observed in patients (Gamelin et al., 1996; Yoshida et al., 1990), so more studies are needed to examine if lower doses of the drug would have an impact on muscle cells. Because total protein levels for phosphorylated proteins were not investigated, it is possible that the observed decreases in the phosphorylation of these signals is due to a decrease in total protein content. Furthermore, due to the decreases in γ‐tubulin levels 48 and 72 h following drug treatment initiation, findings from this study could be attributed to the effect of the chemotherapy drug cocktail on cell death. This is in line with our data showing increased abundance of cleaved caspase 3 and is consistent with studies that show chemotherapy‐induced cell death in cardiomyocytes (Nayak et al., 2013; Priya et al., 2017). However, all data were normalized to their respective γ‐tubulin values. In addition, decreases in the abundance of troponin, tropomyosin and p‐S6K1, and in peptide chain initiation were found 24 h after the initiation of drug treatment, at a time point when γ‐tubulin values did not differ between treatments. Furthermore, myotube nuclei area (Figure 1g,h) was measured and found to not be different between treatments. With regard to our cachexia model, using an in vitro approach does not allow for the elucidation of how these chemotherapy drugs effect the entire body system. Nonetheless, our in vitro model allows for the investigation of mechanisms of direct effects of chemotherapy on myotube protein metabolism and cachexia development in skeletal muscle cells.

In conclusion, we demonstrated specific effects of a chemotherapy drug cocktail on myofibrillar protein abundance and synthesis. These changes occurred in parallel with coordinated alterations in substrate transport, insulin signaling, and markers of mitochondrial abundance. If these data measures are confirmed in vivo, targeting these changes may help to preserve muscle mass during chemotherapy treatment and thus help to increase treatment efficacy, quality of life, and survival of patients.

## DISCLOSURE

The authors declare no conflict of interest.

## AUTHOR CONTRIBUTIONS

SM and OAJA conceived and designed the experiments. SM performed the experiments, analyzed the samples, and drafted the initial version of the manuscript. OAJA reviewed and edited the manuscript. Both authors approved the final version of the manuscript.
